# Ethnic minority status and psychological integration among China's internal migrants: the mediating role of bonding social capital

**DOI:** 10.3389/fpsyg.2026.1769209

**Published:** 2026-07-29

**Authors:** Xu Yuan, Xiaoli Wei, Yong Xie

**Affiliations:** College of Public Administration, Nanjing Agricultural University, Nanjing, China

**Keywords:** bonding social capital, China, ethnic minority migrants, family migration, internal migration, psychological integration

## Abstract

**Objective:**

Despite well-documented socioeconomic disadvantages, ethnic minority migrants in China continue to migrate to urban destinations, presenting the integration paradox. This study aims to examine the relationship between ethnic minority status and psychological integration among China's internal migrants and to explore the mediating pathways, particularly focusing on the mediating role of bonding social capital, thereby unpacking the integration paradox.

**Methods:**

Using data from the 2017 China Migrants Dynamic Survey, ordinary least squares (OLS) regression is *first* employed to examine ethnic differences in psychological integration among China's internal migrants. Bootstrap mediation analysis is then conducted to test the mediating role of bonding social capital, manifested through family migration, household size, and social network interactions.

**Results:**

Ethnic minority migrants suggest significantly higher psychological integration than Han migrants on average. This finding remains robust when using an alternative seven-item measure of psychological integration, and holds consistently across subsamples of rural-to-urban, urban-to-urban, and intra-provincial migrants. Bootstrap mediation results suggest that family migration, household size, and social network interactions each play a significant partial mediating role.

**Conclusion:**

Ethnic minority status is associated with stronger psychological integration through the mediating effects of bonding social capital, manifested through family migration, household size, and social network interactions. Policy implications include reconceptualizing ethnic communities from “structural burdens” to “community resources,” designating family and community as core intervention units, and adopting differentiated policies that address group-specific heterogeneity in resources, migration patterns, and structural challenges.

## 1 Introduction

Navigating ethnic and cultural diversity is a defining challenge for social cohesion in cities globally (Donnell, 2024). While extensive research focuses on international migrants, the integration of internal ethnic minority migrants in large, multi-ethnic states like China remains underexamined, despite its comparable significance for national cohesion and urban policy (Liu et al., 2025). China, with its sizable and expanding ethnic minority population within a society where the Han Chinese (the ethnic majority group in China) constitute the majority ethnic group, offers a compelling context for this investigation. According to China's *Seventh* National Population Census, the ethnic minority population reached 125.67 million in 2020, accounting for approximately 8.89% of the total population. Ethnic minority population has been dispersing notably from their traditional homelands since the turn of the 21st century. According to the 2010 Population Census, ethnic minorities population accounted for 6.81% of the national migrants. By 2017, the Report on China's Migrant Population Development documented that this share had risen to 9.41%. It presents the integration paradox that despite well-documented socioeconomic disadvantages, ethnic minority migrants in China continue to migrate to urban destinations. While this migration fuels urbanization, it reveals a dual reality: it both fosters inter-ethnic exchange and simultaneously raises critical questions about the pathways and depth of inclusion for ethnic minorities (Qiu et al., 2025).

Urban integration is widely conceptualized as a multidimensional construct encompassing economic, social, and psychological dimensions (Hackett, 2003; Oduro, 2024; Tang and Liu, 2019; Yang and Tao, 2021). A robust body of empirical research finds that the urban integration of ethnic minority migrants lags behind that of the Han majority, particularly in terms of objective and measurable socioeconomic outcomes such as employment status and income level (Gan and Zhang, 2021; He, 2019; Xie, 2019). Despite foreseeable difficulties in urban integration, both the absolute number and relative share of ethnic minority migrants continue to grow. Why do minority groups continue to migrate to urban destinations despite persistent marginalization, discrimination, and economic disadvantages? We propose that the integration paradox may be related to relatively higher levels of psychological integration among ethnic minority migrants despite structural disadvantages.

Psychological integration represents the highest level of urban integration, capturing the emotional interaction between individuals and the host society (Harder et al., 2018; Li et al., 2023). Its conceptualization has evolved from a unidirectional focus on identity abandonment (Gordon, 1964) toward a bidirectional understanding (Berry, 1997; Hellgren et al., 2025). Early studies emphasized the process by which individuals gradually come to see themselves as members of the host society (Gordon, 1964), while later frameworks encompassed both migrants' psychological identification with the host society and their perception of being accepted by it (Berry, 1997; Portes and Zhou, 1993). Bollen and Hoyle (1990) conceptualized the psychological dimension of social integration as comprising *two* components: individuals' sense of belonging to a social group and their feeling of being a group member. This emphasis on self-perceived belonging highlights the subjective experience of inclusion in the mainstream urban context (Harder et al., 2018). In contrast, Berry's (1997) concept of “psychological adaptation” focuses primarily on overall mental health and wellbeing. Synthesizing these theoretical insights, this study defines psychological integration as migrants' sense of belonging to, identification with, and perceived acceptance by the urban destination society (Li et al., 2023; Liang, 2020; Yang and Wu, 2021; Zeng et al., 2022). This multidimensional perspective allows us to capture both the affective and cognitive aspects of migrants' psychological ties to the urban destination. This conceptualization anchors psychological integration in the host society, thereby addressing the “adaptation to whom” question implicit in Berry's framework.

Shaped by their intersectional status as both migrants and ethnic minorities, the psychological integration of ethnic minority migrants presents a unique complexity (Bontenbal, 2023; He, 2019). On the *one* hand, migrant status is typically accompanied by institutional barriers rooted in the hukou (household registration) system. As the massive reallocation of labor from agricultural to non-agricultural sectors and from rural to urban areas has played a key role in China's rapid economic growth and urbanization (Cao et al., 2023), the vast majority of migrants hold agricultural hukou (Fan and Li, 2020). Consequently, these migrants face systematic exclusion from urban citizenship rights, including restricted access to healthcare, housing, and education (Zhao et al., 2024). Compounded by urban–rural educational disparities, they often have lower educational attainment and insufficient vocational skills, leading to poorer employment outcomes (Li et al., 2025; Yang and Tao, 2021). Taken together, these institutional constraints hinder not only their economic integration but also their psychological integration.

On the other hand, this study advances a dual-pathway theoretical framework to examine the psychological integration of ethnic minority migrants, compared with their Han counterparts. The framework is grounded in and extends Berry's (1997) *four* acculturation strategies: integration, assimilation, separation, and marginalization. It conceptualizes ethnic minority status as having *two* simultaneous facets: a source of structural constraint and a foundation for community resilience. The structural disadvantage perspective emphasizes that ethnic differences in language, religion, or customs expose minority migrants to discrimination and cultural obstacles (Valentina et al., 2021). These persistent barriers and perceived discrimination can heighten acculturative stress and undermine psychological integration, corresponding to the poorer psychological outcomes associated with separation or marginalization (Berry, 1997; Remennick, 2004). The ethnic resource and resilience perspective delineates a form of bonding social capital, referring to the close, inward-looking ties between family members and within co-ethnic groups, which provide ethnic minority migrants with essential material, informational, and emotional support (Coffé, 2007). This supportive social mechanism can strengthen in-group ties and identity, a core proposition of social identity theory (Tajfel and Turner, 1979). Based on contemporary acculturation theory, such cohesive co-ethnic communities need not lead to social closure; rather, they can serve as a psychological resource that facilitates engagement and integration with mainstream society. For China's ethnic minority migrants, the bonding social capital formed through ethnic clustering, manifested in higher prevalence of family migration, larger household size, and interactions with both co-ethnics and out-group members, jointly facilitates their psychological integration. This is consistent with the positive psychological outcomes associated with successful integration strategies (Elizabeth et al., 2009; Phinney, 1990; Brance et al., 2023).

How, then, does the psychological integration of ethnic minority migrants in China differ from that of their Han counterparts? Could bonding social capital, manifested through family migration, household size, and social network interactions, serve as potential mechanisms facilitating their psychological integration? Answering these questions is essential for explaining the observed integration paradox. This study examines the relationship between ethnic minority status and psychological integration among China's internal migrants and the mediating mechanisms using data from the 2017 China Migrants Dynamic Survey, which employed a stratified, multi-stage, PPS sampling design across provincial-level regions in China. We *first* employ ordinary least squares (OLS) regression to examine ethnic differences in psychological integration among China's internal migrants. Subsequently, we employ bootstrap mediation analysis to test whether bonding social capital, manifested through family migration, household size, and social network interactions, mediates the relationship between ethnic minority status and psychological integration.

Our research makes *two* key contributions. First, the dual-pathway theoretical framework we propose challenges the prevailing view that treats ethnic minority status solely as a structural disadvantage. Building on the recognition of structural disadvantages, we argue that the bonding social capital formed through family and community ties within ethnic minority migrant clusters provides socio-emotional resources that support their psychological integration (Tang and Liu, 2019; Xu and You, 2017). By integrating this framework with empirical mediation analysis, we provide direct evidence for these pathways, offering a more nuanced lens for explaining the integration paradox. Second, this study offers important insights for urban governance in multi-ethnic and multicultural contexts, emphasizing the need for differentiated policies tailored to the specific ethnic resources and community networks of minority migrants to facilitate their urban integration.

## 2 Theory and hypotheses

### 2.1 Literature review

Research on the social and cultural integration of migrants has generated a rich and multifaceted body of literature, with psychological integration emerging as a core dimension. Western scholars have predominantly examined whether migrants integrate into mainstream urban society through perspectives such as identity, the acceptance of and assimilation into local culture, ethnic relations or prejudice, and individual acculturation (Berry, 1997; Goldlust and Richmond, 1974; Portes and Zhou, 1993). Chinese scholars, drawing primarily on theories of social exclusion and social integration, have analyzed the psychological integration of internal migrants through dimensions such as identity, sense of belonging, and perceived acceptance (Guo and Fu, 2020; Liang, 2020; Yang and Wu, 2021; Zeng et al., 2022). Across these diverse lines of inquiry, a growing consensus has emerged that migrants' psychological integration is profoundly shaped by subjective evaluations at both the individual and group levels (Bollen and Hoyle, 1990; Jana et al., 2023). Migrants' sense of belonging, identification, and identity redefinition collectively shape the psychological integration process, which is actively built through everyday engagement with urban environments (Liang, 2020; Zisakou et al., 2023).

Although there is broad consensus on the multidimensional nature of psychological integration, the construction of its indicator system has not yet reached a unified standard. Early attempts, such as that of Bollen and Hoyle (1990), measured the construct through individuals' sense of belonging to a social group and their feeling of being a group member. Casakin et al. (2015) employed indicators such as attachment to the destination and perceived local membership. The China Migrants Dynamic Survey (CMDS) is the most widely used data source for analyzing the urban integration of Chinese internal migrants and its various dimensions (Guo and Fu, 2020; Li et al., 2018). Drawing on this data, Chinese scholars have generally adopted multidimensional frameworks based on identity, sense of belonging, and perceived acceptance to construct indicator systems for psychological integration, with commonly selected indicators including self-identification as a local, willingness to integrate into the local community, attention to changes in the city, and perceived social attitudes (Guo and Fu, 2020; Hu et al., 2024; Liang, 2020; Yang and Wu, 2021; Zeng et al., 2022). Building on this multidimensional approach, Li et al. (2023) developed the Urban Psychological Integration Scale for Chinese Migrant Workers, which comprises *three* dimensions, namely identity identification, social identification, and perceived acceptance, and *nine* items, grounded in the dual aspects of identification and acceptance, thereby providing an important tool for localized measurement.

While a substantial body of research has examined the broader urban integration of ethnic minority migrants in China, literature specifically focusing on their psychological integration remains relatively scarce. These studies consistently show that ethnic minority migrants face disadvantages in employment quality and economic integration compared to the Han majority, often attributed to gaps in human and social capital (Gan and Zhang, 2021; He, 2019). Research employing multidimensional indices also consistently finds minority migrants' overall integration levels to be lower than Han migrants (Cai et al., 2024; Yang and Tao, 2021). The literature has identified a wide range of influencing factors for the urban integration of ethnic minority migrants. These include individual characteristics such as sex, age, hukou status, educational attainment, and marital status (Cai et al., 2024; Yang and Tao, 2021); labor and employment characteristics including employment status, work unit ownership, and wages (Wang and Zhang, 2019); migration characteristics such as migration trajectory, migration range, and migration reasons (Li, 2022); as well as social capital and institutional policies (Han and Xia, 2019; Liang, 2020; Lu et al., 2020). The main explanatory frameworks for the urban integration of migrants emphasize *three* strands: human capital (e.g., educational attainment, vocational skills, and Mandarin proficiency), social capital (e.g., migrants' social networks in destination areas; Chen and Li, 2016; He et al., 2024; Park et al., 2026), and institutional barriers that hinder migrants' integration in host destinations (Lewis et al., 2011; Remennick, 2004).

A substantial body of research has also examined how social capital shapes integration outcomes. Tatarko (2018) finds that bridging social capital (networks connecting individuals to out-groups) indirectly promotes sociocultural adaptation through the integration strategy, while bonding social capital (strong in-group ties) operates through distinct mechanisms. This research aligns with broader theoretical frameworks distinguishing bonding social capital, which provides emotional support and in-group solidarity, from bridging social capital, which facilitates access to novel information and opportunities (Kopren and Westlund, 2021). The protective function of co-ethnic networks has been extensively documented (Li, 2022). Strong in-group ties buffer against discrimination-related stress, provide material and emotional support during early settlement, and foster positive in-group identity, a process theorized by social identity theory (Krüger, 2024; Tajfel and Turner, 1979). Ethnic minority migrants have long demonstrated distinctive patterns of chain migration and community clustering, rooted in shared religious and traditional practices (Xie, 2019). However, the implications of such ethnic clustering for integration remain theoretically contested. While some research emphasizes that dense co-ethnic networks may foster social closure and limit exposure to mainstream opportunity structures (Portes and Zhou, 1993), other studies demonstrate that bonding social capital can coexist with, and even facilitate, broader societal participation, particularly when communities maintain flexible boundaries and institutional supports (Kopren and Westlund, 2021).

Despite significant advances, several theoretical and empirical gaps persist. First, existing perspectives, which primarily address socioeconomic disparities, cannot fully explain the integration paradox: the sustained growth of ethnic minority migration flows persists despite persistent integration disadvantages. Second, the relationship between bonding social capital and psychological integration, specifically whether strong co-ethnic networks facilitate or hinder identification with the host society, remains contested and likely context-dependent. Third, while research increasingly recognizes integration as multidimensional, studies examining interactions among economic, social, and psychological dimensions remain relatively scarce. Addressing these gaps requires a framework that simultaneously accounts for both structural constraints and community-based resources, and that can specify the conditions under which *one* pathway may outweigh the other. This is the central task of the dual-pathway framework proposed in this study.

### 2.2 A dual-pathway theoretical framework

**Figure 1:**
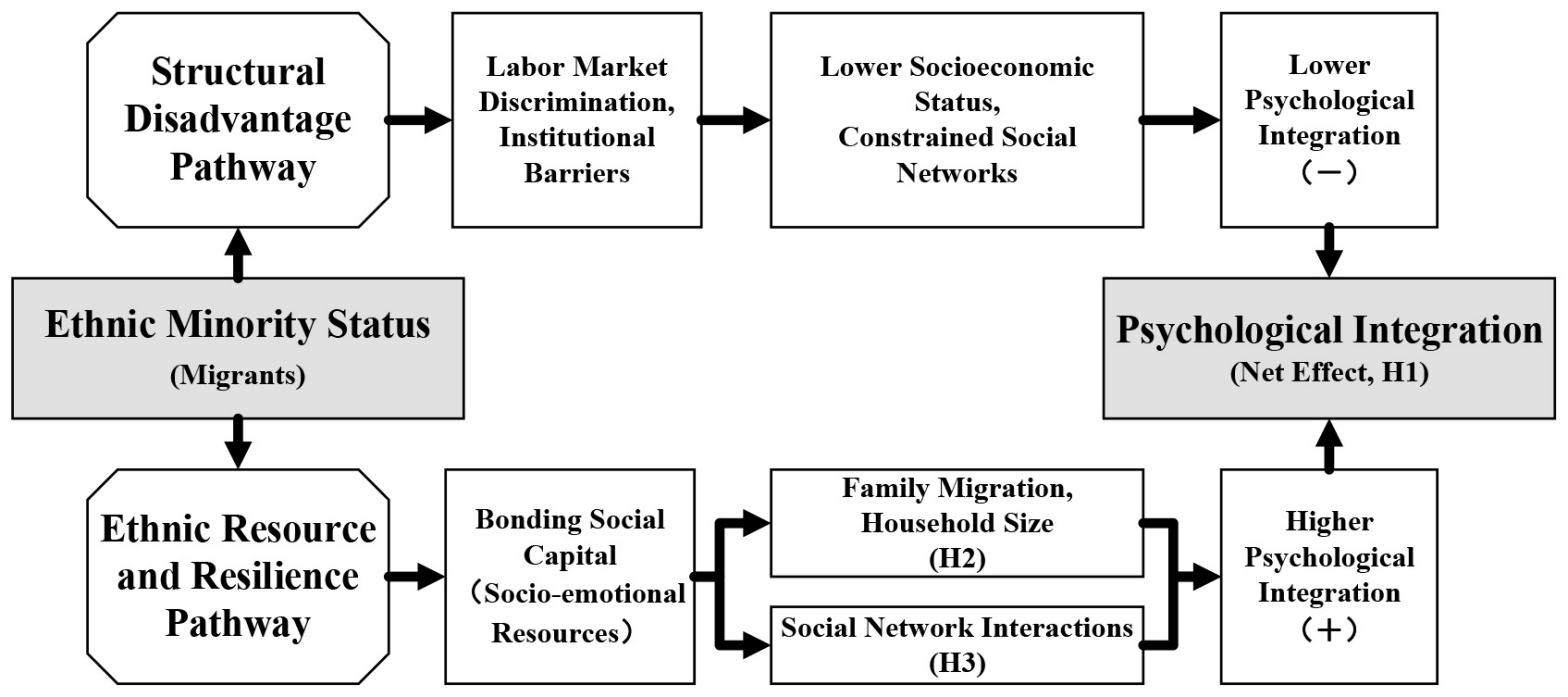
A dual-pathway framework for psychological integration among ethnic minority migrants.

Berry's (1997) acculturation framework provides the foundational conceptual architecture for understanding how minorities navigate intercultural encounters, distinguishing between heritage culture maintenance and intergroup contact to generate *four* acculturation strategies, and further differentiating psychological adaptation from sociocultural adaptation (Berry, 1997; Ward, 1997). Building on this foundation, this study advances a dual-pathway theoretical framework, namely the structural disadvantage pathway and the ethnic resource and resilience pathway, to examine the relationship between ethnic minority status and psychological integration among China's internal migrants (Figure 1).

From the perspective of the structural disadvantage pathway, ethnic minority status is accompanied by disadvantages or differences in language, religious beliefs, customs, and education. These disadvantages expose ethnic minority migrants to labor market discrimination and systemic barriers, which in turn negatively affect their socioeconomic status and social networks, ultimately undermining psychological integration. In our empirical analysis, this pathway is operationalized through indicators such as hukou, educational attainment, employment type, income, and housing type. First, discrimination based on language, religion, or customs occurs both in the labor market and daily life. Non-standard accents or dialects subject ethnic minority migrants to hiring discrimination (Jackson and Denis, 2024; Schulte et al., 2024). Religious practices such as halal dietary restrictions, coordinating prayer times, and Muslim women wearing the hijab often lead to implicit marginalization in institutional settings (Khatatbeh, 2023; Xie, 2019). Similarly, in China, Hui and Uyghur migrants, as Muslim-majority ethnic groups, also encounter cultural barriers and discrimination stemming from their religious practices and distinct customs. Second, compounded by urban–rural disparities in educational resources, ethnic minority migrants generally have lower educational attainment and insufficient vocational skills (Li et al., 2025; Yang and Tao, 2021). These human capital disadvantages further channel them into low-skilled, precarious employment. Taken together, these structural disadvantages constrain career development and social networks, directly eroding migrants' sense of psychological belonging to the urban society (Li et al., 2025). Moreover, these obstacles further trigger internal acculturative stress and identity threat. Berry's (1997) acculturation theory posits that persistent discrimination and cultural barriers can threaten individuals' social identity and heighten acculturative stress. When ethnic minority migrants perceive exclusion from mainstream society, they may tend to adopt a separation strategy (maintaining original culture while rejecting host culture) or a marginalization strategy (rejecting both heritage and host cultures), both of which are typically associated with poorer psychological adaptation.

From the perspective of the ethnic resource and resilience pathway, ethnic minority status can be translated into positive resources for psychological integration through the accumulation of bonding social capital. This study focuses on *two* specific forms of bonding social capital: family-centric bonding social capital (operationalized as higher prevalence of family migration and larger household size) and community-based bonding social capital (operationalized as tighter-knit co-ethnic networks). Notably, these *two* forms of bonding social capital follow a temporal sequence. Family-centric bonding social capital (higher prevalence of family migration and larger household size) tends to be foundational and operates primarily during the early stage of migration, providing immediate emotional security and material support. Community-based bonding social capital (tighter-knit co-ethnic networks), in contrast, develops over time as migrants become more settled, serving as an extended resource for information, collective identity, and cultural continuity. While our cross-sectional data cannot strictly test this temporal ordering, the theoretical distinction aligns with the natural progression of migrant adaptation.

First, family-centric bonding social capital provides ethnic minority migrants with emotional support and stability. A higher prevalence of family migration and larger household size signify tighter family bonds and richer internal resource networks. At the emotional level, family members migrating together can effectively buffer the psychological stress induced by culture shock and discrimination. The companionship of family serves as the *first* line of defense against acculturative stress, helping migrants maintain a positive psychological state when facing external exclusion (Chen et al., 2021; Jorgensen, 2017). At the resource level, larger household size implies a more abundant internal resource network, providing tangible support such as employment information, housing assistance, and childcare. This reduces the uncertainty migrants face in urban life and enhances their sense of control. More fundamentally, as the most basic source of belonging, the family provides a stable emotional anchor for migrants who have yet to establish extensive social connections, preventing them from falling into isolation and helplessness in the city. Notably, this family-centric bonding social capital is associated with a lower risk of separation and primarily serves a protective function (Chen et al., 2021; Jorgensen, 2017). Thus, family-centric bonding social capital operates as an initial, foundational layer of support during the early stages of migration, laying the groundwork for subsequent community-based ties.

Second, community-based bonding social capital emerges over time as migrants become more settled, providing extended resources and further strengthening their foundation for psychological integration through collective identity and mutual support networks. The ethnic clustering and chain migration patterns of ethnic minority migrants have formed tight-knit co-ethnic networks, whose functions are manifested in *three* dimensions. First, information and opportunity transmission: co-ethnic networks serve as primary channels for disseminating crucial information such as employment opportunities, housing resources, and policy changes, compensating for the information disadvantages arising from insufficient bridging social capital (Li, 2022; Xu and You, 2017). Second, collective identity reinforcement: frequent interactions with co-ethnics strengthen positive in-group identity, enabling ethnic minority migrants to draw psychological strength from collective belonging when facing external discrimination (Krüger, 2024). Third, cultural continuity: co-ethnic networks provide ethnic minority migrants with spaces to maintain their language, religion, and customs, allowing them to gradually adapt to urban life without abandoning their heritage culture. This cultural continuity helps alleviate acculturative stress and facilitates psychological adaptation.

However, there is persistent theoretical debate regarding the impact of community-based bonding social capital on psychological integration. *One* strand of scholarship emphasizes its risks, arguing that such capital may inhibit the formation of bridging social capital, leading to social closure and alienation from mainstream culture (Portes and Zhou, 1993; Xiang, 2021). This is a “separation” outcome in Berry's (1997) terms. In contrast, another line of research highlights its resource function, viewing psychological integration as a dynamic, non-zero-sum process (Panicacci, 2021). From this perspective, maintaining heritage culture and participating in mainstream culture can be mutually reinforcing, not oppositional (Panicacci, 2019; Ryder et al., 2000). This perspective suggests that bonding and bridging social capital are not mutually exclusive; rather, under conditions of strong co-ethnic identity, bonding social capital can not only provide a secure foundation for belonging but also serve as a psychological resource that actively encourages the formation of bridging social capital. In other words, community-based bonding social capital among ethnic minority migrants does not necessarily hinder mainstream integration; rather, it may empower them to extend their social networks beyond ethnic boundaries, thereby enhancing psychological integration (Chen et al., 2021; Sun et al., 2024; Zhang et al., 2023). This study tests this pathway *via* mediation analysis (higher prevalence of family migration, larger household size, and social network interactions with both co-ethnics and out-group members).

Given the proposed integration paradox, this study posits that the bonding social capital of China's ethnic minority migrants primarily assumes a resource function. It can both sustain a resilient form of separation and serve as a springboard from separation to an integration strategy (Portes and Zhou, 1993; Tang and Liu, 2019). This strategic shift implies that migrants maintain their heritage cultural identity while establishing positive connections with mainstream society, which is the core feature of high-level psychological integration. Specific minority groups provide illustrative examples of this strategic transformation through the development of ethnic niche economies, such as the Hui people with their Lanzhou hand-pulled noodles and the Uyghur people with their Xinjiang-style barbecue (Tang and Liu, 2019). This economic model is not only a product of family and community bonding social capital but also serves as an incubator for bridging social capital. When ethnic economies become successfully embedded in the urban industrial chain, migrants' opportunities for contact with mainstream society naturally increase, and bonding social capital transforms from an internal support resource into a bridge for accessing external resources. At this point, the in-group sense of belonging that migrants derive from bonding social capital becomes a psychological resource for seeking recognition within mainstream society and constructing a dual identity. This dual identity is both the core feature of the integration strategy and a manifestation of high-level psychological integration.

Thus, ethnic minority status simultaneously activates *two* pathways. (1) The structural disadvantage pathway: the disadvantages or differences in language, religious beliefs, and education associated with ethnic minority status expose them to employment discrimination and institutional barriers. Labor market discrimination and institutional barriers lead to lower socioeconomic status and constrained social networks, thereby undermining psychological integration. (2) The ethnic resource and resilience pathway: ethnic minority migrants exhibit distinct patterns of family migration and ethnic clustering. Family-centric and community-based bonding social capital not only provide migrants with emotional support, resource networks, and in-group identity, but also transform these capitals into psychological resources for engaging with mainstream society, thereby facilitating psychological integration. Within this pathway, the *two* forms of bonding capital play distinct yet complementary roles: family-centric capital provides foundational emotional security and stability, while community-based capital builds upon this foundation to foster collective identity, social participation, and connections with mainstream society. They jointly facilitate the psychological integration of ethnic minority migrants. Therefore, the psychological integration of ethnic minority migrants is the net outcome of the joint operation of the structural disadvantage pathway and the ethnic resource and resilience pathway. This study proposes the following hypothesis:

*Hypothesis 1 (H1)*: Ethnic minority status is positively related to psychological integration among internal migrants in China.

The ethnic resource and resilience pathway operates primarily through *two* levels: the family level and the community level. At the family level, a higher prevalence of family migration and larger household size provide migrants with emotional support and stability, thereby enhancing their psychological integration. At the community level, bonding social capital provides migrants with emotional support, resource networks, and in-group identity, enabling them to extend their social networks beyond ethnic boundaries and form interactions with both co-ethnics and out-group members, thereby strengthening psychological integration (Chen et al., 2021; Sun et al., 2024; Zhang et al., 2023). Accordingly, the following hypotheses are proposed:

*Hypothesis 2 (H2)*: The higher prevalence of family migration and larger household size among ethnic minority migrants mediate the relationship between ethnic minority status and psychological integration.
*Hypothesis 3 (H3)*: Social network interactions with both co-ethnics and out-group members mediate the relationship between ethnic minority status and psychological integration.

## 3 Methods

### 3.1 Data

This study uses data from the 2017 China Migrants Dynamic Survey (CMDS), administered by the former National Health and Family Planning Commission (NHFPC). The primary purpose of the CMDS is to systematically collect information on the migration patterns, living conditions, and socioeconomic integration of China's internal migrant population to inform policy-making. The survey was administered through face-to-face interviews conducted by trained enumerators, with participation being voluntary. Respondents were assured of data confidentiality and provided informed consent prior to the interview. The survey employed a stratified, multi-stage, PPS sampling design across provincial-level regions in China to ensure national representativeness. While the CMDS covers both urban and rural destination areas, the overwhelming flow of internal migrants is toward urban socioeconomic systems. The essence of this migration is a transition from a rural social system into an urban socioeconomic system (characterized by non-agricultural economies), defined by a shift from agricultural to non-agricultural employment, even as migrants' residences span urban, suburban, or rural settings. Thus, “local” or “urban” here operationally refers to these broader urban destinations. The target population comprised mainland Chinese citizens aged 15 or above who had resided locally for at least 1 month without local household registration (hukou), thereby excluding international migrants. After excluding observations with missing core data, the final analytical sample includes 169,478 internal migrants, of whom 15,953 (9.41%) are from ethnic minority groups.

### 3.2 Variables

#### 3.2.1 Dependent variable: psychological integration

Informed by theoretical conceptualizations of psychological integration (Berry, 1997; Bollen and Hoyle, 1990; Gordon, 1964; Hellgren et al., 2025; Li et al., 2023; Portes and Zhou, 1993) and established measurement practices in studies using CMDS data (Guo and Fu, 2020; Liang, 2020; Yang and Wu, 2021; Zeng et al., 2022), we operationalize psychological integration as a three-dimensional construct comprising affective belonging, identification, and perceived acceptance. Affective belonging is measured by “I like the city/place where I am currently living” (Item 1) and “I pay attention to the changes in the city/place where I currently live” (Item 2); identification is measured by “I am willing to integrate into the local community and become *one* of them” (Item 3) and “I feel that I am already a local” (Item 4); perceived acceptance is measured by “I feel that local residents are willing to accept me as *one* of them” (Item 5) and “I feel that locals look down on outsiders” (Item 6, reverse-coded). All items are rated on a 4-point Likert scale ranging from 1 (strongly disagree) to 4 (strongly agree).

The measurement model is evaluated by examining internal reliability, convergent validity, and discriminant validity. First, the KMO measure of sampling adequacy is 0.838, and Bartlett's test of sphericity is significant (*p* < 0.001), indicating that the data are suitable for factor analysis. Second, the Cronbach's alpha coefficient for the overall scale is 0.794, exceeding the recommended threshold of 0.70 (Gliem and Gliem, 2003), indicating good internal consistency. For the *three* subdimensions, alpha values are 0.776 (affective belonging), 0.606 (identification), and 0.446 (perceived acceptance). The relatively low alpha for the two-item dimensions is consistent with known psychometric properties, as Cronbach's alpha tends to underestimate reliability for scales with few items (Eisinga et al., 2013). The lower alpha for perceived acceptance is consistent with known methodological challenges associated with reverse-coded items (Weijters et al., 2013). Third, confirmatory factor analysis (CFA) is conducted to test the three-factor structure. The model demonstrates excellent fit: χ^2^(6) = 2593.78, CFI = 0.993, TLI = 0.981, SRMR = 0.017, RMSEA = 0.050 [90% CI (0.049, 0.052)]. All standardized factor loadings are significant (*p* < 0.001), ranging from 0.325 to 0.894 (see Table 1). Composite reliability (CR) for the overall scale is 0.858, and average variance extracted (AVE) is 0.522, both meeting the recommended thresholds (Hair et al., 2019). Fourth, to assess discriminant validity, we compare the three-factor model with a one-factor model. The three-factor model significantly outperforms the one-factor model (Δχ^2^ = 15,709.92, Δdf = 3, *p* < 0.001), supporting the distinctiveness of the *three* dimensions (see Table 2). The high correlations among the latent factors (ranging from 0.712 to 0.892) further suggest they reflect a shared higher-order construct.

**Table 1:** Construct reliability and validity.

Dimension	Items	Loading	Cα	CR	AVE
Affective belonging	Item 1	0.798	0.776	0.777	0.635
	Item 2	0.796			
Identification	Item 3	0.545	0.606	0.638	0.478
	Item 4	0.812			
Perceived acceptance	Item 5	0.894	0.446	0.576	0.453
	Item 6	0.325			
Psychological integration scale			0.794	0.858	0.522

**Table 2:** Results of confirmatory factor analysis.

Models	χ^2^	df	χ^2^/df	RMSEA	CFI	TLI	SRMR
One-factor model	18,303.70	9	2,033.74	0.110	0.947	0.912	0.035
Three-factor model	2,593.78	6	432.30	0.050	0.993	0.981	0.017

Finally, following standard practice in integration research (Liang, 2020; Yang and Wu, 2021; Zeng et al., 2022), we construct a composite index by *first* normalizing each item using min-max scaling as shown in Equation (1), then averaging the *six* standardized items, and finally multiplying by 100 to obtain a 0–100 scale. Higher scores indicate stronger psychological integration.


$$\begin{array}{l} x_{i}^{*}=\frac{x_{i}-x_{\min}}{x_{\max}-x_{\min}} \end{array}$$ (1)

#### 3.2.2 Independent variables: ethnic minority

The primary independent variable is the ethnicity of the migrant population. It is operationalized in *two* complementary ways. First, we constructed a binary indicator distinguishing the ethnic majority from all minorities. Second, to account for the substantial socioeconomic and cultural heterogeneity across minority groups in China, we created a detailed categorical variable. This variable isolates the *nine* largest minority groups by population: Mongol, Manchu, Hui, Tibetan, Zhuang, Uyghur, Miao, Yi, and Tujia. All remaining minority individuals were combined into an “Other Minority” category to ensure statistical reliability, as their group sample sizes were too small for stable, separate estimation. Ethnic groups with fewer than 360 observations (e.g., < 0.21% of the minority sample) were combined into an “Other Minority” category to ensure statistical reliability. In all analyses, the Han majority serves as the reference group. The demographic distribution of these ethnic categories in our sample is presented in Table 3.

**Table 3:** Distribution of ethnic groups.

Ethnicity		N	Population Share (%)
Han		153,525	90.59
Ethnic minority	Mongol	772	0.46
	Manchu	1,168	0.69
	Hui	3,507	2.07
	Tibetan	1,547	0.91
	Zhuang	2,107	1.24
	Uyghur	927	0.55
	Miao	1,143	0.67
	Yi	712	0.42
	Tujia	1,120	0.66
	Other Ethnic Minorities	2,950	1.74
Ethnic minority		15,953	9.41

#### 3.2.3 Control variables

Following established literature (Cai et al., 2024; Li et al., 2022; Wang and Zhang, 2019; Yang and Tao, 2021), this study controls for variables across *four* dimensions: demographic characteristics, migration characteristics, employment characteristics, and family characteristics. Demographic characteristics include sex (male = 1, female = 0), age, hukou (agricultural = 1, otherwise = 0), marital status, and education. Marital status (married = 1, otherwise = 0) is derived from a single-choice question. Due to data constraints, we cannot differentiate between intra-ethnic and inter-ethnic marriages. Education is measured as the highest degree completed, categorized into *four* levels (primary school or below = 1, junior high school = 2, senior high school =3, college or above = 4) from a single-choice item. Migration characteristics consist of migration duration, residence count, and migration range. Migration duration (in years) is a continuous measure of the current length of stay in the destination, calculated as the difference between the survey year and the self-reported month and year of move moving to the current city (a fill-in-the-blank item). Residence count is the self-reported total number of cities lived in for 1 month or longer (fill-in-the-blank). Migration range captures the geographical scope of the current move, based on a single-choice question with *three* options: within-city across counties, intra-provincial across cities, and inter-provincial. The survey was conducted in urban areas, thus capturing migration flows to urban destinations. Employment characteristics are measured by the ownership type of the current work unit, constructed from a single-choice question and categorized into *five* types (state-owned/collective, individual business, private enterprise, foreign-invested/joint venture, other), with state-owned/collective as the reference category. Family characteristics include household income and current housing type. Household income is the log-transformed monthly household income per capita. Current housing type, from a single-choice question, is recoded into *three* categories: other housing types (reference), purchased non-commercial housing, and purchased commercial housing. Table 4 presents the descriptive statistics. The full wording of all survey questions, response options, and detailed operationalization is provided in Supplementary Table S4-1. Descriptive statistics by ethnicity (Han vs. minority) are provided in Supplementary Table S4-2.

**Table 4:** Descriptive statistics of variables.

Variable		Mean	SD
Psychological integration		74.609	15.367
Ethnic minority		0.094	0.292
Sex		0.517	0.500
Age		36.639	11.047
Hukou		0.780	0.415
Marital status		0.813	0.390
Education	Primary school or below	0.170	0.376
	Junior high school	0.437	0.496
	Senior high school	0.219	0.414
	College or above	0.174	0.379
Ownership of work unit	State-owned or collective	0.070	0.256
	Individual business	0.341	0.474
	Private enterprise	0.229	0.420
	Foreign-invested or joint venture	0.034	0.180
	Other	0.326	0.469
Residence count		1.973	1.901
Migration range	Within-city across counties	0.177	0.382
	Intra-provincial across cities	0.330	0.470
	Inter-provincial	0.493	0.500
Migration duration		6.310	6.062
Current housing type	Other housing types	0.748	0.434
	Non-commercial housing purchased	0.038	0.191
	Commercial housing purchased	0.214	0.410
Household income		7.625	0.650

#### 3.2.4 Mediating variables

To empirically examine the *two* proposed mediating mechanisms, stronger family migration and denser co-ethnic networks, we operationalized them using the following measures and variables, drawing on the survey data.

First, family migration. This is a binary variable constructed from the survey question, “What is the primary reason for your current move?” (single choice). Responses such as “working as an employee” and “business/self-employment” were categorized as “economic/development-oriented migration” (coded 0). Responses including “moving with family”, “marriage”, “joining relatives”, “birth”, and “caring for elderly/children” were categorized as “family-oriented migration” (coded 1). This dichotomy distinguishes between instrumental and affective migration motivations, though it may oversimplify some nuanced reasons.

Second, household size. This variable is derived from the mandatory question, “Number of co-residing family members,” and is used as a continuous indicator of household scale and potential familial support.

Third, social network interactions. This index measures the breadth of an individual's formal organizational participation. It is constructed by summing affirmative responses (Yes = 1) to seven binary (Yes/No) questions regarding participation since 2016 in activities organized by: (a) a labor union, (b) a volunteers association, (c) an alumni association, (d) a fellow townspeople association, (e) a hometown chamber of commerce, (f) any other organized activities, and (g) whether they have made suggestions or overseen affairs in their workplace, community, or village. The resulting count (range 0–7) serves as a proxy for the level of engagement in structured local social activities. While this measure captures the breadth of participation, it does not account for interaction frequency or quality, which remains a limitation.

### 3.3 Empirical strategy

To address the research objectives, we examine the association between ethnicity and the psychological integration of migrants using the full sample. We estimate *two* separate sets of ordinary least squares (OLS) regressions, corresponding to the *two* alternative operationalizations of the key independent variable. The *first* model employs the binary ethnicity indicator. The specification is presented in Equation (2):


$$\begin{array}{l} Psychology_{i}=\alpha_{0}+\alpha_{1}Minority_{i}+\alpha_{2}Control_{i}+\varepsilon_{i} \end{array}$$ (2)

In Equation (2), *Psychology*_*i*_ represents the psychological integration score (0–100) of migrant i. *Minority*_*i*_ is a binary indicator of ethnic minority status. *Control*_*i*_ represents a series of control variables. ε_*i*_ is the error term.

The *second* model employs the detailed categorical ethnicity variable, as specified in Equation (3):


$$\begin{array}{l} Psychology_{i}=\beta_{0}+\beta_{1}Category_{i}+\beta_{2}Control_{i}+\varepsilon_{i} \end{array}$$ (3)

Here, *Category*_*i*_ denotes a set of dummy variables representing the largest specific minority groups and an “Other Minority” category, with the Han majority serving as the reference group. *Control*_*i*_ is identical to that in Equation (2). Since the dependent variable is continuous, both equations are estimated using OLS.

To formally test the mediating pathways proposed in Hypotheses 2 and 3, we conduct a bootstrap mediation analysis with 1,000 resamples. This approach directly estimates the indirect effect of ethnic minority status on psychological integration through each proposed mediator and provides bias-corrected percentile confidence intervals. An indirect effect is considered statistically significant if its 95% confidence interval does not contain zero. The *three* mediators, namely family migration (binary), household size (continuous), and social network interactions (count), are each estimated separately using the same set of control variables as in the baseline regressions.

## 4 Results

### 4.1 Benchmark analysis

The baseline OLS regression results are presented in Table 5 (using a binary ethnicity indicator) and Table 6 (disaggregated by specific ethnic group). Both models control for a set of variables spanning demographic characteristics, migration characteristics, employment characteristics, and family characteristics. Table 5 shows that, after controlling for other factors, ethnic minority migrants report a statistically significant higher level of psychological integration compared to the Han majority. The coefficient of 1.019 (*p* < 0.001) indicates an average positive association between minority status and the integration index.

**Table 5:** The effect of ethnicity on the psychological integration of migrants.

Variable	β	SE	t	*p*	95% CI	
					LL	UL
Ethnic minority	1.019	0.127	8.030	0.000	0.770	1.268
Sex	0.017	0.074	0.230	0.814	−0.128	0.163
Age	0.083	0.004	20.670	0.000	0.076	0.091
Hukou	−1.964	0.094	−20.910	0.000	−2.148	−1.780
Marital status	0.480	0.101	4.770	0.000	0.283	0.677
Education						
Junior high school	1.236	0.113	10.980	0.000	1.015	1.457
Senior high school	2.677	0.131	20.480	0.000	2.421	2.933
College or above	4.498	0.150	29.910	0.000	4.203	4.793
Ownership of work unit						
Individual business	0.168	0.154	1.090	0.276	−0.134	0.470
Private enterprise	−1.180	0.157	−7.530	0.000	−1.487	−0.873
Foreign-invested or joint venture	−2.608	0.234	−11.120	0.000	−3.067	−2.148
Other	0.232	0.154	1.500	0.134	−0.071	0.534
Residence count	−0.222	0.022	−9.930	0.000	−0.266	−0.178
Migration range						
Intra-provincial across cities	−1.610	0.106	−15.230	0.000	−1.817	−1.403
Inter-provincial	−4.105	0.103	−40.000	0.000	−4.306	−3.904
Migration duration	0.216	0.007	32.930	0.000	0.203	0.229
Current housing type						
Non-commercial housing purchased	5.752	0.192	29.930	0.000	5.376	6.129
Commercial housing purchased	5.105	0.094	54.500	0.000	4.921	5.288
Household income	−0.111	0.063	−1.750	0.079	−0.235	0.013
_cons	72.066	0.554	130.020	0.000	70.980	73.152
R^2^	0.084					
F	833.467					

**Table 6:** The effect of specific ethnic groups on migrants' psychological integration.

Variable	β	SE	t	*p*	95% CI	
					LL	UL
Ethnic minority category						
Mongol	0.100	0.530	0.190	0.850	−0.939	1.140
Manchu	0.808	0.428	1.890	0.059	−0.031	1.648
Hui	1.953	0.264	7.390	0.000	1.435	2.470
Tibetan	1.185	0.357	3.320	0.001	0.485	1.885
Zhuang	−0.173	0.320	−0.540	0.590	−0.801	0.455
Uyghur	8.062	0.508	15.860	0.000	7.066	9.059
Miao	−0.960	0.455	−2.110	0.035	−1.853	−0.068
Yi	0.029	0.548	0.050	0.957	−1.046	1.104
Tujia	−0.702	0.461	−1.520	0.128	−1.605	0.202
Other ethnic minorities	0.602	0.279	2.160	0.031	0.055	1.149
Sex	−0.004	0.074	−0.050	0.960	−0.149	0.141
Age	0.085	0.004	21.070	0.000	0.077	0.093
Hukou	−1.929	0.094	−20.530	0.000	−2.113	−1.744
Marital status	0.489	0.101	4.860	0.000	0.292	0.686
Education						
Junior high school	1.295	0.114	11.400	0.000	1.073	1.518
Senior high school	2.755	0.132	20.890	0.000	2.496	3.013
College or above	4.578	0.151	30.220	0.000	4.281	4.875
Ownership of work unit						
Individual business	0.176	0.154	1.150	0.252	−0.125	0.478
Private enterprise	−1.122	0.157	−7.160	0.000	−1.429	−0.815
Foreign-invested or joint venture	−2.555	0.234	−10.900	0.000	−3.015	−2.096
Other	0.217	0.154	1.410	0.160	−0.085	0.519
Residence count	−0.218	0.022	−9.790	0.000	−0.262	−0.174
Migration range						
Intra-provincial across cities	−1.666	0.106	−15.760	0.000	−1.873	−1.459
Inter-provincial	−4.085	0.103	−39.760	0.000	−4.287	−3.884
Migration duration	0.216	0.007	32.860	0.000	0.203	0.228
Current housing type						
Non-commercial housing purchased	5.731	0.192	29.810	0.000	5.355	6.108
Commercial housing purchased	5.079	0.094	54.200	0.000	4.896	5.263
Household income	−0.074	0.063	−1.160	0.244	−0.198	0.051
_cons	71.636	0.556	128.850	0.000	70.547	72.726
R^2^	0.085					
F	575.393					

This aggregate result, however, conceals considerable heterogeneity across ethnic groups, as detailed in Table 6. Disaggregating ethnicity reveals that the average positive association is largely driven by a subset of specific minorities. Notably, Uyghur (β = 8.062, *p* < 0.001), Hui (β = 1.953, *p* < 0.001), Tibetan (β = 1.185, *p* = 0.001), and Manchu (β = 0.808, *p* = 0.059) minorities report significantly higher psychological integration scores. By contrast, the Miao minority reports a significantly lower level of integration (β = −0.960, *p* = 0.035). No statistically significant differences from the Han majority are observed for Mongol, Zhuang, Yi, or Tujia migrants. Therefore, while the pooled estimate provides initial support for Hypothesis H1, the apparent “minority advantage” is not uniform; it represents an average of markedly divergent outcomes, with the positive aggregate effect driven primarily by certain groups.

The estimated effects of the control variables are consistent across specifications and align with theoretical expectations. Educational attainment demonstrates a strong positive gradient; higher education levels are associated with substantially greater psychological integration. Migration duration is positively related to psychological integration (β = 0.216, *p* < 0.001), whereas a greater number of previous residences and a broader migration range (both intra-provincial and inter-provincial) correlate negatively. Current housing type, specifically, owning commercial or non-commercial housing is among the strongest positive predictors. Holding a rural hukou remains a significant institutional barrier to integration. Compared to working in state-owned or collective units, employment in private enterprises or foreign-invested/joint ventures is associated with significantly lower psychological integration. Age and marital status show significant positive associations with psychological integration.

In summary, the benchmark analysis establishes a significant average association between ethnic minority status and higher psychological integration. Crucially, it further reveals that this aggregate effect is characterized by substantial internal heterogeneity, with specific groups driving the positive result. The analysis also confirms the vital roles of human capital, long-term and stable local settlement, favorable institutional status, and employment type in shaping migrants' psychological integration.

### 4.2 Robust analysis

To assess whether the selection of indicators and weighting scheme influence the baseline results, we construct an alternative index by incorporating a *seventh* indicator: “intention to transfer hukou.” This item is derived from the survey question: “If you meet the local settlement requirements, are you willing to transfer your household registration to this city?” (single-choice question; 1 = No, 2 = Undecided, 3 = Yes). While hukou transfer intention may reflect institutional considerations, in the Chinese context it fundamentally signals long-term settlement commitment and psychological internalization of local membership. This expanded index retains equal weighting across all components. Regression results using this alternative measure are presented in Table 7. The coefficient for ethnic minority remains positive and statistically significant at the 1% level (β = 1.681). The stability of this estimate confirms that the observed “ethnic advantage” in psychological integration is robust to the inclusion of this commitment-based dimension, thereby strengthening the validity of our core conclusion.

**Table 7:** Robustness check: alternative seven-item index including hukou transfer intention.

Variable	β	SE	t	*p*	95% CI	
					LL	UL
Ethnic minority	1.681	0.131	12.880	0.000	1.426	1.937
Control variables	Yes					
R^2^	0.084					
F	839.649					

Control variables are consistent with the baseline regression. Full results are available in the Supplementary Material

### 4.3 Endogeneity analysis

To address potential endogeneity arising from self-selection on observable characteristics, we supplement our analysis with Propensity Score Matching (PSM). This method mitigates selection bias by constructing a counterfactual sample in which ethnic minority migrants are matched with Han migrants who are statistically similar across all observed control variables. Balance checks indicate that the matching procedure achieves good balance for the vast majority of control variables, suggesting that observable differences between the *two* groups are effectively accounted for. We estimated the Average Treatment Effect on the Treated (ATT) using *three* matching algorithms, nearest-neighbor (1:1 and 1:4) and kernel matching, with bootstrap standard errors. The results, presented in Table 8, consistently show a positive and statistically significant ATT. For instance, the ATT from 1:1 nearest-neighbor matching is 0.947 [SE = 0.256, 95% CI (0.524, 1.370), *p* < 0.001]. This suggests that, after accounting for self-selection based on observed factors, the psychological integration score of ethnic minority migrants remains significantly higher by about 0.95 points than that of their observationally equivalent Han counterparts.

**Table 8:** Average treatment effects from propensity score matching.

Matching	ATT	Bootstrap Std. Err	z	*p*	95% CI
Nearest neighbor matching (1:1)	0.947	0.256	4.390	0.000	[0.524, 1.370]
Nearest neighbor matching (1:4)	0.993	0.162	6.130	0.000	[0.676, 1.311]
Radius matching	0.997	0.156	6.380	0.000	[0.691, 1.303]

It is important to note, however, that PSM only addresses selection bias on observable characteristics. Bias from unobserved factors may still persist. Therefore, while this analysis suggests that the observed association is not driven solely by observable differences, the results should still be interpreted as a robust correlation rather than a causal effect. Future research using panel data or instrumental variable approaches is needed to more rigorously address potential unobserved confounding.

### 4.4 Mediation analysis

To formally test the mediating pathways proposed in Hypotheses 2 and 3, we conduct a bootstrap mediation analysis with 1,000 resamples, following the framework of Preacher and Hayes (2008). This approach directly estimates the indirect effect of ethnic minority status on psychological integration through each proposed mediator and provides bias-corrected percentile confidence intervals, which do not rely on the assumption of normality. An indirect effect is considered statistically significant if its 95% confidence interval does not contain zero. The results, presented in Table 9, indicate that all *three* indirect pathways had bootstrap 95% confidence intervals that excluded zero, confirming the significance of these effects.

**Table 9:** Bootstrap mediation results for psychological integration.

Path	Indirect effect	SE	95% CI	p	Result
Ethnic minority → family migration → psychological integration	0.024	0.005	[0.014, 0.035]	0.000	Supported
Ethnic minority → household size → psychological integration	0.003	0.001	[0.001, 0.006]	0.054	Supported
Ethnic minority → social network interactions → psychological integration	0.052	0.0121	[0.029, 0.076]	0.000	Supported

Bootstrap with 1,000 resamples. 95% confidence intervals are bias-corrected percentile intervals. Following Preacher and Hayes (2008), indirect effects are considered statistically significant when the 95% confidence interval does not contain zero

The bootstrap mediation results revealed significant indirect effects for all *three* pathways. Specifically, the indirect effect of ethnic minority status on psychological integration through family migration was 0.024 [95% CI (0.014, 0.035)], suggesting that the higher propensity for family migration among ethnic minority migrants is associated with their stronger psychological integration. The indirect effect through household size was 0.003 [95% CI (0.001, 0.006)], indicating that larger household size also serves as a significant mediator, albeit with a smaller magnitude. Notably, while the *p*-value for household size was 0.054 (slightly above the conventional threshold of 0.05), the bootstrap confidence interval [0.001, 0.006] did not contain zero, confirming its statistical significance. The indirect effect through social network interactions was 0.052 [95% CI (0.029, 0.076)], showing that greater breadth of participation in formal organizational activities likewise mediates the association between ethnic minority status and psychological integration. Collectively, these findings provide robust support for Hypotheses 2 and 3. They demonstrate that bonding social capital, manifested through family migration, household size, and social network interactions, serves as a key mediating mechanism linking ethnic minority status to stronger psychological integration.

### 4.5 Heterogeneity analysis

The above analysis shows that the psychological integration of ethnic minority migrants is generally significantly higher than that of Han migrants on average. Next, this section further examines the heterogeneous effects of ethnic factors on psychological integration across different migration trajectories and migration ranges.

China's rural-urban dual hukou (household registration) system constitutes a key institutional factor in integration. Based on hukou type, the migration trajectory of migrants with agricultural hukou is defined as moving from rural to urban areas (henceforth “R-U”), while the migration trajectory of migrants with non-agricultural hukou is defined as moving from *one* urban area to another urban area (henceforth “U-U”). Compared with U-U migrants, R-U migrants face not only institutional barriers in accessing social welfare such as housing, employment, education, and medical care, but also additional obstacles related to their lifestyles and cultural adaptation. We divided the sample into U-U migrants and R-U migrants to conduct heterogeneity analysis. As can be seen from Table 10, whether in the rural-urban or the urban-urban subsample, the psychological integration level of ethnic minority migrants is significantly higher than that of Han migrants.

**Table 10:** The effect of ethnicity on psychological integration by migration trajectories and ranges.

Migration trajectories												
	R-U						U-U					
Variable	β	SE	t	p	95% CI		β	SE	t	p	95% CI	
					LL	UL					LL	UL
Ethnic minority	1.018	0.139	7.340	0.000	0.746	1.289	1.146	0.291	3.940	0.000	0.576	1.715
Control variables	Yes						Yes					
N	132,126						37,352					
R^2^	0.070						0.072					
F	554.553						160.843					
**Variable**	**Migration ranges**											
	**Intra-provincial**						**Inter-provincial**					
Ethnic minority	1.971	0.157	12.510	0.000	1.662	2.279	−0.530	0.205	−2.580	0.010	−0.933	−0.128
Control variables	Yes						Yes					
N	85,931						83,547					
R^2^	0.049						0.090					
F	260.624						485.155					

Control variables are consistent with the baseline regression. Full results are available in the Supplementary Material, where a table of contents is provided to guide readers

Beyond migration direction, China's vast territory makes migration distance a critical factor in integration, with inter-provincial boundaries serving as an effective proxy for distance. The administrative jurisdiction in China follows a hierarchical structure from largest to smallest: province, city, and county. In the full sample, ethnic minority migrants are primarily intra-provincial migrants, whereas the proportions of Han migrants moving intra-provincially and inter-provincially are roughly equal. The benchmark regression controlled for migration range, and the coefficient was statistically significant. For heterogeneity analysis, we divided the sample into intra-provincial migrants (including within-city across-county migration and intra-provincial across-city migration) and inter-provincial migrants. According to Table 10, among the intra-provincial migrants, the psychological integration level of ethnic minority migrants is still significantly higher than that of Han migrants. However, among the inter-provincial migrants, the psychological integration level of ethnic minority migrants is significantly lower than that of Han migrants. This indicates that for inter-provincial migrants, the ethnic resource and resilience pathway is insufficient to fully counteract the structural disadvantages intensified by long-distance migration. A table of contents is provided at the beginning of the Supplementary Material to guide readers to the relevant sections.

## 5 Discussion

This study aims to explain the integration paradox by examining the role of bonding social capital in the psychological integration of China's ethnic minority migrants. Our findings suggest that, on average, ethnic minority migrants report higher levels of psychological integration than their Han counterparts, primarily through the accumulation of bonding social capital. This finding challenges the structural disadvantage thesis of minority integration (Remennick, 2004) and aligns with the ethnic resource and resilience thesis, which posits that co-ethnic networks can provide socio-emotional resources that buffer against structural disadvantages (Chen et al., 2021; Portes and Zhou, 1993). At the same time, our results suggest substantial heterogeneity in psychological integration across different ethnic minority groups (see Table 6). This pattern suggests a critical refinement to the ethnic resource and resilience thesis: the potency of bonding social capital and social network interactions in fostering psychological resilience is not inherent to minority status *per se*, but is likely moderated by group-specific factors.

The average ethnic advantage is primarily driven by *four* specific minority groups: the Manchu (0.69%), Hui (2.07%), Tibetan (0.91%), and Uyghur (0.55%). Together, these *four* groups account for approximately half of the total ethnic minority migrant population. Notably, these groups share key characteristics: large population sizes, well-established patterns of chain migration and community clustering, and the presence of distinctive ethnic economies. Their bonding social capital not only buffers against structural disadvantages, but also actively facilitates their integration into urban society (Tang and Liu, 2019; Xu and You, 2017). Consistent with the ethnic resource and resilience pathway advanced in our theoretical framework, greater family migration propensity, larger household size, and more social network interactions serve as psychological resources that facilitate their psychological integration (Jorgensen, 2017).

For migrants from other major minority groups, such as the Zhuang (1.24%), Tujia (0.66%), Mongol (0.46%), and Yi (0.42%), their levels of psychological integration show no significant difference from those of Han migrants. While these groups also possess substantial populations, their bonding social capital, formed through chain migration and community clustering, appears to be primarily effective in compensating for structural disadvantages, bringing their self-perceived psychological integration to a level comparable with, but not surpassing, that of the Han majority (Remennick, 2004; Xie, 2019). This finding resonates with the view that ethnic clustering can be a compensatory mechanism for upward mobility (Portes and Zhou, 1993), suggesting that bonding social capital operates along a continuum, from passive buffering to active enhancement, depending on group-specific characteristics. This continuum reflects the varying degrees to which co-ethnic networks are able to translate internal support into external opportunities.

Only Miao migrants (0.67%) exhibit levels of psychological integration that are significantly lower than those of their Han counterparts. This unique disadvantage can be attributed to the precarious social and institutional position of the Miao. Historically practicing a migratory, swidden agricultural lifestyle, many Miao migrants, particularly those who have moved spontaneously, face severe structural vulnerabilities (Zheng and Zhang, 2021). They often lack formal documentation and are not integrated into local governance systems, which excludes them from state poverty alleviation programs and public services. Consequently, they are trapped in a marginalized status characterized by economic hardship, land disputes, and a lack of political rights. Their social networks tend to be highly insular, limiting their interaction with the broader community and reinforcing their position as cultural outsiders in urban settings (Diamond, 1988). This deep-seated institutional exclusion and social closure overwhelm the protective effects of their co-ethnic networks, preventing them from converting bonding social capital into a sense of belonging to the host society (Berry, 1997). This case exemplifies the structural disadvantage pathway in our dual-pathway framework, illustrating how, under conditions of extreme marginalization, even co-ethnic networks may prove insufficient to foster psychological integration (Xiang, 2021).

Several limitations should be noted. First, constrained by the operationalization limitations of pre-collected data from the CMDS, we were unable to adopt a more comprehensive measure of psychological integration. Instead, we relied on *six* indicators capturing affective belonging, identification, and perceived acceptance, a measurement approach widely adopted in studies using the CMDS data (Guo and Fu, 2020; Hu et al., 2024; Liang, 2020; Yang and Wu, 2021). Each dimension was measured using only *two* items, which inherently yields lower reliability estimates (Eisinga et al., 2013). The perceived acceptance dimension further includes a reverse-coded item, known to introduce method effects that attenuate inter-item correlations (Weijters et al., 2013). Future research should validate these findings using more comprehensive scales with multi-item balanced measures for each dimension. Second, the estimated associations in this study do not fully address endogeneity issues arising from omitted variables. Although we employed propensity score matching to mitigate observable selection bias, unobserved confounding factors cannot be completely ruled out. Future studies should seek appropriate instrumental variables to address endogeneity, thereby strengthening the rigor of the research design. Third, the finding that ethnic minority migrants report higher psychological integration than their Han counterparts represent an average effect. Future research would benefit from distinguishing the types of resources across different ethnic minority groups and then, through appropriately designed mediation analyses, exploring which specific co-ethnic or community resources can mitigate structural disadvantages and support psychological integration. That is, future work should examine the conditions under which bonding social capital mechanisms operate.

## 6 Conclusion

This study investigates the differences in psychological integration across different ethnic groups of domestic migrants, drawing on the 2017 CMDS data. Our analysis suggests that, on average, ethnic minority migrants report higher levels of psychological integration than their Han counterparts. This finding remains robust when using an alternative measure of psychological integration and holds across subsamples of R-U, U-U, and intra-provincial migrants. Second, bootstrap mediation results provide empirical support for the ethnic resource and resilience pathway, showing that bonding social capital, manifested through family migration, household size, and social network interactions, significantly mediates the relationship between ethnic minority status and psychological integration. Third, our findings offer a potential explanation for the integration paradox: the sustained growth of ethnic minority migration is associated with the psychological buffer of tight-knit community structures, which may reduce the impact of objective socioeconomic disadvantages. These results support the ethnic resource and resilience pathway within our dual-pathway framework, highlighting the role of bonding social capital as manifested through family and community networks.

This study offers several policy implications for facilitating the urban integration of ethnic minority migrants. First, it calls for a reconceptualization of ethnic minority communities from “structural burdens” to “community resources,” recognizing them as important psychological buffers and incubators of social capital. Policy approaches should therefore move beyond a narrow focus on compensating for disadvantages toward a balanced strategy that leverages community-based strengths. Second, “family” and “community” should be treated as core units of policy intervention. At the family level, policies should encourage family migration by reducing institutional barriers to reunification and considering household size in housing and education policies. At the community level, support should be directed toward both intra-ethnic mutual aid organizations and cross-ethnic activities that facilitate bridging social capital, promoting positive interaction between bonding and bridging social capital. Third, a differentiated policy evaluation system should be established to account for heterogeneity across ethnic minority groups. Policy formulation should attend to differences in resource endowments, migration patterns, and community networks, implementing targeted interventions such as legal assistance, social security coverage, and community integration support for groups facing specific structural challenges.

## Data Availability

The raw data supporting the conclusions of this article will be made available by the authors, without undue reservation.
