# Supplementary material for: Ethnic minority status and psychological integration among China's internal migrants: the mediating role of bonding social capital
Source: Front Psychol. 2026 Jul 29;17:1769209. doi: 10.3389/fpsyg.2026.1769209 (PMC13461610; doi:10.3389/fpsyg.2026.1769209)
Supplement: Supplementary file 1 [file Supplementary_file_1.docx]

**Supplemental material**

TABLE S4-1. Variable names, survey questions, and operational definitions.

| Variable | Survey Question | Note/Definition |
| --- | --- | --- |
| Psychological integration | To what extent do you agree with the statement: "I like the city/place where I am currently living"?  1 = Strongly disagree; 2 = Disagree; 3 = Agree; 4 = Strongly agree |  |
|  | Do you agree with the statement, "I pay attention to the changes in the city/place where I currently live"?  1 = Strongly disagree; 2 = Disagree; 3 = Agree; 4 = Strongly agree |  |
|  | To what extent do you agree with the statement: "I am willing to integrate into the local community and become one of them"?  1 = Strongly disagree; 2 = Disagree; 3 = Agree; 4 = Strongly agree |  |
|  | To what extent do you agree with the statement: "I feel that I am already a local"?  1 = Strongly disagree; 2 = Disagree; 3 = Agree; 4 = Strongly agree |  |
|  | To what extent do you agree with the statement: "I feel that local residents are willing to accept me as one of them"?  1 = Strongly disagree; 2 = Disagree; 3 = Agree; 4 = Strongly agree |  |
|  | Do you agree with the statement, "I feel that locals look down on outsiders"?  1 = Strongly disagree; 2 = Disagree; 3 = Agree; 4 = Strongly agree | Reverse-coded |
| Ethnicity | Ethnicity (single-choice question):  1 Han  2 Mongol  3 Manchu  4 Hui  5 Tibetan  6 Zhuang  7 Uyghur  8 Miao  9 Yi  10 Tujia  …… |  |
| Sex | Sex (single-choice question):  1 Male  2 Female | Male=1, Female=0 |
| Age | Year of Birth (fill-in-the-blank). |  |
| Hukou | Residence registration type (single-choice question):  1 Agricultural  2 Non-agricultural  3 Agricultural-to-resident  4 Non-agricultural-to-resident | Agricultural=1, Other=0 |
| Marital status | Marital status (single-choice question):  1 Unmarried  2 First marriage  3 Remarriage  4 Divorce  5 Widowed  6 Cohabitation | Married (First marriage/Remarriage) =1, Other =0 |
| Education | Education (single-choice question):  1 Primary school or below  2 Junior high school  3 Senior high school  4 College or above | 1= Primary school or below  2= Junior high school  3= Senior high school  4= College or above |
| Ownership of work unit | What is the ownership type of your current employer/work unit? (single-choice question)  01 State-owned or collective,  02 Individual business,  03 Private enterprise,  04 Foreign-invested or joint venture,  05 Other | 1= State-owned or collective  2= Individual business  3= Private enterprise  4= Foreign-invested or joint venture  5= Other |
| Residence count | In total, how many cities have you ever lived in (including your current residence)? (fill-in-the-blank) | Refers to moves across district/county borders for one month or longer for purposes such as work or living, excluding business trips, visiting relatives, tourism, medical treatment, etc. |
| Migration Range | Scope of the current move (single-choice question):  1 Within-city across counties  2 Intra-provincial across cities  3 Inter-provincial | 1= Within-city across counties  2= Intra-provincial across cities  3= Inter-provincial |
| Migration duration | Date of the current move: Month & Year (fill-in-the-blank).  Continuous residence in the inflow destination is counted as a single spell; temporary absences of less than one month do not constitute a new migration event. | Survey date minus move date, expressed in years (rounded to one decimal place). |
| Current housing type | Which of the following best describes your current housing?  01 Employer-provided housing (excluding workplace accommodation)  21 Renting private housing-entire unit  22 Renting private housing-shared unit  03 Public rental housing provided by the government  04 Commercial housing purchased  05 Affordable housing purchased  06 Property-rights-restricted housing purchased  07 Living in borrowed accommodation  08 Workplace accommodation  09 Self-built housing  10 Other informal dwellings | 1= Other housing types  2= Non-commercial housing purchased (05, 06)  3= Commercial housing purchased (04) |
| Household income | What was your household's average monthly total income over the past year? (fill-in-the-blank) | Log (monthly Household income /Household size) |
| Family migration  (Reason for migration) | Primary reason for the current move (single-choice question):  01 Work/Employment  02 Business/Self-employment  03 Moving with family (Taking care of the elderly or children)  04 Marriage  05 Joining relatives or friends  06 Study or training | 1= Family-oriented migration (03, 04, 05)  0= Economic/development-oriented migration (01, 02, 06) |
| Household size | How many people are in your household, including yourself, your spouse, your children (including those living locally, in your hometown, or elsewhere, but excluding those who have established separate households), and other family members currently living with you? (fill-in-the-blank) | Counting |
| Social network interactions | Since 2016, have you participated in any activities organized by a labor union in your current locality? (single-choice question)  Yes=1; No=0 | Sum of seven binary items (range 0–7) |
|  | Since 2016, have you participated in any activities organized by a volunteer association in your current locality?  Yes=1; No=0 |  |
|  | Since 2016, have you participated in any activities organized by an alumni association in your current locality?  Yes=1; No=0 |  |
|  | Since 2016, have you participated in any activities organized by a fellow townspeople association in your current locality?  Yes=1; No=0 |  |
|  | Since 2016, have you participated in any activities organized by a hometown chamber of commerce in your current locality?  Yes=1; No=0 |  |
|  | Since 2016, have you participated in any other organized activities (not listed above) in your current locality?  Yes=1; No=0 |  |
|  | Since 2016, have you made suggestions to or overseen the affairs of your workplace, community, or village?  Yes=1; No=0 |  |
| Co-ethnic network density | Who do you spend the most time with socially in your local area during your leisure time (excluding customers and relatives)?  1 Fellow townspeople/villagers (household registration has been moved to the local area)  2 Fellow townspeople/villagers (household registration remains in the hometown)  3 Fellow townspeople/villagers (household registration has been moved to a region other than the local area or hometown)  4 Other local residents  5 Other non-local residents  6 Rarely socialize with others | 1=Interaction primarily with fellow townspeople/villagers  0=Others |
| Intention to transfer Hukou | If you meet the local settlement requirements, are you willing to transfer your household registration to this city? (single-choice question)  1 No  2 Undecided  3 Yes | 1=No  2=Undecided  3=Yes |

TABLE S4-2 Descriptive statistics by ethnicity (Han vs. minority).

| Variable | | Definition and Measurement | Ethnic Minority Migrants（N=15953） | | Han Migrants（N=153525） | | Mean Difference |
| --- | --- | --- | --- | --- | --- | --- | --- |
|  |  |  | Mean | SD | Mean | SD |  |
| Psychological integration | | Composite index (0–100) | 75.107 | 15.828 | 74.558 | 15.317 | 0.550^***^ |
| Ethnic minority | | Ethnic minority = 1, Han = 0 | 1.000 | 0.000 | 0.000 | 0.000 |  |
| Sex | | Male = 1, Female = 0 | 0.498 | 0.500 | 0.519 | 0.500 | -0.021^***^ |
| Age | | Age in 2017 | 35.374 | 11.177 | 36.771 | 11.025 | -1.397^***^ |
| Hukou | | Agricultural=1, Other=0 | 0.831 | 0.375 | 0.774 | 0.418 | 0.057^***^ |
| Marital Status | | Married = 1, Otherwise = 0 | 0.754 | 0.431 | 0.819 | 0.385 | -0.064^***^ |
| Education | Primary school or below | 0.309 | 0.462 | 0.155 | 0.362 | -0.153^***^ | 0.153^***^ |
|  | Junior high school | 0.385 | 0.487 | 0.442 | 0.497 | 0.057^***^ | -0.057^***^ |
|  | Senior high school | 0.167 | 0.373 | 0.225 | 0.417 | 0.058^***^ | -0.058^***^ |
|  | College or above | 0.139 | 0.346 | 0.178 | 0.382 | 0.039^***^ | -0.039^***^ |
| Ownership of work unit | State-owned or collective | 0.073 | 0.260 | 0.070 | 0.255 | -0.003 | 0.003 |
|  | Individual business | 0.299 | 0.458 | 0.346 | 0.476 | 0.046^***^ | -0.046^***^ |
|  | Private enterprise | 0.210 | 0.407 | 0.231 | 0.422 | 0.022^***^ | -0.022^***^ |
|  | Foreign-invested or joint venture | 0.024 | 0.152 | 0.035 | 0.183 | 0.011^***^ | -0.011^***^ |
|  | Other | 0.394 | 0.489 | 0.318 | 0.466 | -0.076^***^ | 0.076^***^ |
| Residence count | | Count. | 1.878 | 1.776 | 1.983 | 1.913 | -0.105^***^ |
| Migration Range | Within-city, across counties | 0.218 | 0.413 | 0.173 | 0.378 | -0.045^***^ | 0.045^***^ |
|  | Intra-provincial, across cities | 0.429 | 0.495 | 0.319 | 0.466 | -0.110^***^ | 0.110^***^ |
|  | Inter-provincial | 0.352 | 0.478 | 0.508 | 0.500 | 0.156^***^ | -0.156^***^ |
| Migration duration | Survey date minus move date, expressed in years (rounded to one decimal place). | 6.080 | 5.954 | 6.334 | 6.073 | 0.254^***^ | -0.254^***^ |
| Current housing type | Other types of housing | 0.805 | 0.396 | 0.742 | 0.438 | -0.063^***^ | 0.063^***^ |
|  | Self-purchased non-commercial housing (e.g., subsidized or informal property) | 0.036 | 0.187 | 0.038 | 0.192 | 0.002 | -0.002 |
|  | Self-purchased commercial housing | 0.159 | 0.365 | 0.220 | 0.414 | 0.062^***^ | -0.062^***^ |
| Household income | | In logarithm (Original unit: Yuan) | 7.457 | 0.712 | 7.642 | 0.640 | -0.185^***^ |

TABLE 7 Robustness check: alternative seven-item index including hukou transfer intention.

|  | β | SE | t | p | 95% CI | |
| --- | --- | --- | --- | --- | --- | --- |
|  |  |  |  |  | LL | UL |
| Ethnic minority | 1.681 | 0.131 | 12.880 | 0.000 | 1.426 | 1.937 |
| Gender | -0.183 | 0.076 | -2.430 | 0.015 | -0.331 | -0.035 |
| Age | 0.055 | 0.004 | 13.280 | 0.000 | 0.047 | 0.063 |
| Hukou | -3.265 | 0.096 | -34.090 | 0.000 | -3.452 | -3.077 |
| Marital status | 0.612 | 0.103 | 5.930 | 0.000 | 0.410 | 0.815 |
| Education |  |  |  |  |  |  |
| Junior high school | 1.464 | 0.115 | 12.760 | 0.000 | 1.239 | 1.689 |
| Senior high school | 3.454 | 0.133 | 25.930 | 0.000 | 3.193 | 3.715 |
| College or above | 5.980 | 0.153 | 39.080 | 0.000 | 5.680 | 6.280 |
| Ownership of work unit |  |  |  |  |  |  |
| Individual business | -0.253 | 0.157 | -1.610 | 0.107 | -0.561 | 0.054 |
| Private enterprise | -0.932 | 0.160 | -5.830 | 0.000 | -1.245 | -0.618 |
| Foreign-invested or joint venture | -2.022 | 0.243 | -8.330 | 0.000 | -2.498 | -1.547 |
| Other | 0.710 | 0.157 | 4.510 | 0.000 | 0.401 | 1.018 |
| Residence count | -0.305 | 0.024 | -12.850 | 0.000 | -0.352 | -0.259 |
| Migration range |  |  |  |  |  |  |
| Intra-provincial across cities | -0.266 | 0.106 | -2.500 | 0.012 | -0.475 | -0.058 |
| Inter-provincial | -2.065 | 0.103 | -19.970 | 0.000 | -2.268 | -1.862 |
| Migration duration | 0.261 | 0.007 | 39.060 | 0.000 | 0.247 | 0.274 |
| Current housing type |  |  |  |  |  |  |
| Non-commercial housing purchased | 5.561 | 0.193 | 28.820 | 0.000 | 5.183 | 5.940 |
| Commercial housing purchased | 4.111 | 0.094 | 43.810 | 0.000 | 3.927 | 4.295 |
| Household income | 0.265 | 0.064 | 4.120 | 0.000 | 0.139 | 0.392 |
| _cons | 66.045 | 0.565 | 116.810 | 0.000 | 64.937 | 67.153 |
| R^2^ | 0.084 | | | | | |
| F | 839.649 | | | | | |

TABLE 10 The effect of ethnicity on psychological integration by migration trajectories and ranges.

|  | Migration trajectories | | | | | | | | | | | |
| --- | --- | --- | --- | --- | --- | --- | --- | --- | --- | --- | --- | --- |
|  | R-U | | | | | | U-U | | | | | |
|  | β | SE | t | p | 95% CI | | β | SE | t | p | 95% CI | |
|  |  |  |  |  | LL | UL |  |  |  |  | LL | UL |
| Ethnic minority | 1.018 | 0.139 | 7.340 | 0.000 | 0.746 | 1.289 | 1.146 | 0.291 | 3.940 | 0.000 | 0.576 | 1.715 |
| Gender | 0.018 | 0.084 | 0.210 | 0.832 | -0.148 | 0.183 | -0.028 | 0.155 | -0.180 | 0.857 | -0.331 | 0.275 |
| Age | 0.082 | 0.005 | 17.200 | 0.000 | 0.072 | 0.091 | 0.092 | 0.008 | 12.200 | 0.000 | 0.077 | 0.107 |
| Marital status | 0.370 | 0.117 | 3.180 | 0.001 | 0.142 | 0.599 | 0.904 | 0.205 | 4.420 | 0.000 | 0.503 | 1.305 |
| Education |  |  |  |  |  |  |  |  |  |  |  |  |
| Junior high school | 1.210 | 0.117 | 10.330 | 0.000 | 0.980 | 1.439 | 1.549 | 0.336 | 4.610 | 0.000 | 0.890 | 2.208 |
| Senior high school | 2.762 | 0.143 | 19.320 | 0.000 | 2.482 | 3.043 | 2.498 | 0.343 | 7.280 | 0.000 | 1.826 | 3.170 |
| College or above | 4.642 | 0.176 | 26.400 | 0.000 | 4.297 | 4.987 | 4.246 | 0.358 | 11.870 | 0.000 | 3.545 | 4.947 |
| Ownership of work unit |  |  |  |  |  |  |  |  |  |  |  |  |
| Individual business | 0.059 | 0.194 | 0.300 | 0.762 | -0.322 | 0.440 | 0.117 | 0.265 | 0.440 | 0.660 | -0.403 | 0.636 |
| Private enterprise | -1.399 | 0.200 | -7.010 | 0.000 | -1.790 | -1.008 | -0.756 | 0.259 | -2.920 | 0.004 | -1.264 | -0.248 |
| Foreign-invested or joint venture | -2.916 | 0.290 | -10.050 | 0.000 | -3.485 | -2.348 | -1.874 | 0.443 | -4.230 | 0.000 | -2.743 | -1.006 |
| Other | 0.132 | 0.196 | 0.670 | 0.501 | -0.252 | 0.516 | 0.192 | 0.258 | 0.740 | 0.456 | -0.314 | 0.698 |
| Residence count | -0.204 | 0.021 | -9.550 | 0.000 | -0.246 | -0.162 | -0.294 | 0.046 | -6.360 | 0.000 | -0.384 | -0.203 |
| Migration range |  |  |  |  |  |  |  |  |  |  |  |  |
| Intra-provincial across cities | -1.478 | 0.122 | -12.110 | 0.000 | -1.717 | -1.238 | -2.086 | 0.212 | -9.850 | 0.000 | -2.501 | -1.671 |
| Inter-provincial | -3.914 | 0.116 | -33.620 | 0.000 | -4.143 | -3.686 | -4.749 | 0.216 | -22.000 | 0.000 | -5.172 | -4.326 |
| Migration duration | 0.229 | 0.007 | 31.350 | 0.000 | 0.215 | 0.244 | 0.166 | 0.013 | 12.320 | 0.000 | 0.139 | 0.192 |
| Current housing type |  |  |  |  |  |  |  |  |  |  |  |  |
| Non-commercial housing purchased | 5.969 | 0.226 | 26.450 | 0.000 | 5.526 | 6.411 | 5.085 | 0.358 | 14.190 | 0.000 | 4.382 | 5.787 |
| Commercial housing purchased | 5.360 | 0.113 | 47.320 | 0.000 | 5.138 | 5.582 | 4.481 | 0.171 | 26.150 | 0.000 | 4.146 | 4.817 |
| Household income | -0.219 | 0.071 | -3.080 | 0.002 | -0.359 | -0.080 | 0.323 | 0.131 | 2.460 | 0.014 | 0.066 | 0.580 |
| _cons | 70.879 | 0.616 | 115.000 | 0.000 | 69.671 | 72.087 | 69.142 | 1.108 | 62.400 | 0.000 | 66.970 | 71.314 |
| N | 132126 | | | | | | 37352 | | | | | |
| R^2^ | 0.070 | | | | | | 0.072 | | | | | |
| F | 554.553 | | | | | | 160.843 | | | | | |
|  | Migration ranges | | | | | | | | | | | |
|  | Intra-provincial | | | | | | Inter-provincial | | | | | |
|  | β | SE | t | p | 95% CI | | β | SE | t | p | 95% CI | |
|  |  |  |  |  | LL | UL |  |  |  |  | LL | UL |
| Ethnic minority | 1.971 | 0.157 | 12.510 | 0.000 | 1.662 | 2.279 | -0.530 | 0.205 | -2.580 | 0.010 | -0.933 | -0.128 |
| Gender | -0.231 | 0.103 | -2.240 | 0.025 | -0.434 | -0.029 | 0.249 | 0.106 | 2.350 | 0.019 | 0.041 | 0.457 |
| Age | 0.062 | 0.006 | 11.270 | 0.000 | 0.052 | 0.073 | 0.105 | 0.006 | 18.270 | 0.000 | 0.093 | 0.116 |
| Hukou | -2.459 | 0.125 | -19.720 | 0.000 | -2.704 | -2.215 | -1.253 | 0.147 | -8.520 | 0.000 | -1.541 | -0.965 |
| Marital status | 0.496 | 0.140 | 3.540 | 0.000 | 0.221 | 0.770 | 0.441 | 0.146 | 3.020 | 0.003 | 0.155 | 0.728 |
| Education |  |  |  |  |  |  |  |  |  |  |  |  |
| Junior high school | 1.185 | 0.157 | 7.520 | 0.000 | 0.876 | 1.494 | 1.350 | 0.151 | 8.920 | 0.000 | 1.053 | 1.647 |
| Senior high school | 2.133 | 0.182 | 11.740 | 0.000 | 1.777 | 2.489 | 3.166 | 0.182 | 17.380 | 0.000 | 2.809 | 3.523 |
| College or above | 3.257 | 0.208 | 15.670 | 0.000 | 2.850 | 3.664 | 5.621 | 0.219 | 25.700 | 0.000 | 5.192 | 6.049 |
| Ownership of work unit |  |  |  |  |  |  |  |  |  |  |  |  |
| Individual business | -0.431 | 0.205 | -2.100 | 0.036 | -0.833 | -0.028 | 0.596 | 0.234 | 2.550 | 0.011 | 0.138 | 1.054 |
| Private enterprise | -0.560 | 0.212 | -2.640 | 0.008 | -0.975 | -0.145 | -1.725 | 0.235 | -7.330 | 0.000 | -2.187 | -1.264 |
| Foreign-invested or joint venture | -1.459 | 0.394 | -3.700 | 0.000 | -2.232 | -0.687 | -3.150 | 0.317 | -9.930 | 0.000 | -3.772 | -2.528 |
| Other | -0.002 | 0.205 | -0.010 | 0.992 | -0.404 | 0.399 | 0.261 | 0.235 | 1.110 | 0.266 | -0.199 | 0.721 |
| Residence count | -0.102 | 0.032 | -3.190 | 0.001 | -0.165 | -0.039 | -0.282 | 0.024 | -11.570 | 0.000 | -0.329 | -0.234 |
| Migration duration | 0.177 | 0.009 | 19.290 | 0.000 | 0.159 | 0.195 | 0.239 | 0.009 | 26.470 | 0.000 | 0.221 | 0.256 |
| Current housing type |  |  |  |  |  |  |  |  |  |  |  |  |
| Non-commercial housing purchased | 4.827 | 0.236 | 20.420 | 0.000 | 4.364 | 5.290 | 7.620 | 0.323 | 23.570 | 0.000 | 6.986 | 8.253 |
| Commercial housing purchased | 4.379 | 0.122 | 35.790 | 0.000 | 4.139 | 4.619 | 6.328 | 0.147 | 43.000 | 0.000 | 6.040 | 6.617 |
| Household income | 0.010 | 0.090 | 0.110 | 0.914 | -0.166 | 0.185 | -0.331 | 0.087 | -3.810 | 0.000 | -0.502 | -0.161 |
| _cons | 72.029 | 0.773 | 93.180 | 0.000 | 70.513 | 73.544 | 67.731 | 0.790 | 85.740 | 0.000 | 66.182 | 69.279 |
| N | 85931 | | | | | | 83547 | | | | | |
| R^2^ | 0.049 | | | | | | 0.090 | | | | | |
| F | 260.624 | | | | | | 485.155 | | | | | |
